# Dichotomous roles of ACBD3 in NSCLC growth and metastasis

**DOI:** 10.1038/s41388-025-03360-w

**Published:** 2025-04-06

**Authors:** Xiaochao Tan, Chao Wu, Priyam Banerjee, Shike Wang, Derrick L. Cardin, Yuting Xu, Chad J. Creighton, William K. Russell

**Affiliations:** 1 Department of Medicine, Tulane University School of Medicine, Louisiana Cancer Research Center, New Orleans, LA USA; 2 Department of Thoracic/Head and Neck Medical Oncology, The University of Texas-MD Anderson Cancer Center, Houston, TX USA; 3 Bio-Imaging Resource Center, The Rockefeller University, New York, NY USA; 4 Department of Medicine and Dan L Duncan Cancer Center, Baylor College of Medicine, Houston, TX USA; 5 Department of Biochemistry and Molecular Biology, The University of Texas Medical Branch, Galveston, TX USA

**Keywords:** Lung cancer, Golgi

## Abstract

Lung cancer continues to be the leading cause of cancer-related deaths globally. Unraveling the regulators behind lung cancer growth and its metastatic spread, along with understanding the underlying mechanisms, is crucial for developing novel and effective therapeutic strategies. While much research has focused on identifying potential oncogenes or tumor suppressors, the roles of certain genes can vary depending on the context and may even exhibit contradictory effects. In this study, we demonstrate that acyl-CoA binding domain containing 3 (ACBD3), a Golgi resident protein, promotes primary lung cancer growth by recruiting phosphatidylinositol (PI)-4-kinase IIIβ (PI4KB) to the Golgi, thereby enhancing oncogenic secretion in chromosome 1q-amplified lung cancer cells. Conversely, in chromosome 1q-diploid lung cancer cells, ACBD3 acts as a suppressor of lung cancer metastasis by inhibiting the NOTCH signaling pathway and reducing cancer cell motility. This highlights the intricacy of cancer progression and cautions against simplistic approaches targeting individual oncogenes for cancer therapy.

## Introduction

Non-Small Cell Lung Cancer (NSCLC) is the most prevalent form of lung cancer and has remained the leading cause of cancer-related deaths for decades [1]. The primary subtypes of NSCLC include lung adenocarcinoma (LUAD), lung squamous cell carcinoma (LUSC), and large-cell carcinoma. A lack of comprehensive understanding of this deadly disease has limited the success of current therapeutic approaches, including immunotherapy and targeted therapies.

Cancer development unfolds across a protracted, multistep journey, encompassing initiation, promotion, and metastasis. At each stage, distinct genetic and epigenetic alterations occur, activating disparate pathways. It is widely recognized that cancer cells transition from a phase of rapid proliferation to one of high motility, initiating metastatic dissemination [2]. Consequently, the same gene or pathway may assume varying, even contrasting roles, contingent upon the cancer’s developmental stage. For instance, miR-200 exhibits diverse functions in different metastatic cascades. While the miR-200 family serves as a well-established suppressor of epithelial-mesenchymal transition (EMT), impeding cancer motility and spread [3, 4], it promotes metastatic colonization in the advanced stages of cancer metastasis, partly through the regulation of metastasis-suppressive protein secretion [5]. Similarly, the TGF-β signaling pathway initially plays a tumor-suppressive role by impeding cell cycle progression and fostering apoptosis. However, once a tumor is initiated, TGF-β induces EMT, fostering tumor invasiveness and metastasis [6]. Likewise, the autophagy pathway serves to prevent tumor initiation by triggering apoptotic processes that eliminate precancerous cells [7]. However, for late-stage tumors exposed to stress, it transforms into a dynamic process of degradation and recycling that fuels cancer metastasis [8]. Hence, comprehensively elucidating the dichotomous and contextual function of these cancer regulators is not only critical for understanding the dynamics of cancer progression but also essential for guiding the development of efficient therapeutic strategies.

The Golgi apparatus serves as a crucial hub for protein secretion, orchestrating cargo sorting and trafficking [9]. Over the past decade, dysregulated Golgi proteins have emerged as pivotal regulators of cancer progression [10–17]. Previous studies have revealed that Golgi resident proteins are frequently upregulated in lung cancer due to gene amplification or transcriptional/post-transcriptional activation, fostering cancer development by augmenting the secretion of pro-tumorigenic factors [12–17]. Golgi localized PI-4-phosphate (PI4P), which is generated by two Golgi-dedicated enzymes phosphatidylinositol (PI)-4-kinase IIIβ (PI4KB) and IIα (PI4K2A), plays a critical role in vesicle biogenesis and secretion [18]. Our previous investigations have shown frequent amplification of PI4KB in LUAD, with PI4KB-driven PI4P synthesis enhancing secretion and accelerating LUAD progression [16]. Unlike PI4K2A, which employs palmitoylation for membrane insertion [19], PI4KB lacks a membrane-targeting motif and instead relies on interactions with other Golgi-resident proteins, such as ACBD3 [20], for its Golgi localization. However, its mechanisms of Golgi targeting remain poorly studied in the context of cancer. Acyl-CoA-binding domain-containing protein 3 (ACBD3), also known as Golgi resident protein GCP60, is a multifunctional protein primarily localized in the Golgi apparatus. ACBD3 contains an acyl-CoA binding domain, which may facilitate acyl-CoA binding and transport, and a GOLD (Golgi Dynamics) domain that mediates its Golgi localization [21]. To date, several ACBD3-interacting proteins have been identified, including Golgi resident proteins [20, 22–24], viral proteins [20, 25, 26], and others [27–29]. Through interactions with diverse partners, ACBD3 has been implicated in a range of cellular processes, including steroidogenesis [30], lipid metabolism [27, 31] and transportation [28, 32], viral replication [20, 22, 26, 33], Huntington’s disease [34], and human cancers.

Overexpression of ACBD3 has been identified as a prognostic marker in several human cancers [35–37]. In breast and gastric cancers, ACBD3 promotes tumorigenesis by activating the Wnt/β-catenin signaling pathway and facilitating G1-to-S cell cycle transition [35, 37], respectively. Recent studies have demonstrated that ACBD3 is involved in the regulation of PKA activation [38, 39] and ferroptosis [40], both of which play important roles in cancer progression. However, the role of ACBD3 in the Golgi secretory pathway and in lung cancer progression remains poorly understood. In this study, we investigated the function of ACBD3 in NSCLC progression and uncovered an unexpected dichotomous role of ACBD3 in tumor growth and metastasis. These findings may caution against the indiscriminate development of therapeutic strategies targeting ACBD3 in NSCLC.

## Materials and Methods

### Ethics approval and consent to participate

All mouse studies were conducted in accordance with the guidelines and regulations approved by the Institutional Animal Care and Use Committee (IACUC) at Tulane School of Medicine. Approval for the mouse experiments was obtained from Tulane IACUC (protocol 2004). This publication does not include human subjects or images of human research participants.

### Animal husbandry

Mice received standard care and were euthanized according to the established protocols of the IACUC. Mice were randomly assigned to different groups prior to tumor cell injection. For subcutaneous tumor generation, male nu/nu mice (5–10 mice per group) were subcutaneously injected with 1 × 10^6^ human lung cancer cells. Orthotopic lung tumors were generated by intrathoracic injection of 1 × 10^6^ human lung cancer cells into male nu/nu mice. For intravenous injection model, 1 × 10^5^ 344P-RFP or 1 × 10^6^ A549-RFP cells were injected into the tail vein of male 129/sv or nu/nu mice, respectively. The investigators were not blinded to group allocation during the experiment or outcome assessment.

### Reagents

We purchased SYBR Green, fetal bovine serum (FBS), Dulbecco’s minimal essential medium (DMEM), RPMI Media 1640, Alexa Fluor-tagged secondary antibodies, and DAPI from Life Technologies; puromycin from InvivoGene; paraformaldehyde from Electron Microscopy Sciences; Transwell and Matrigel-coated Boyden chambers from BD Biosciences; G418 from Corning; We obtained shRNAs against human ACBD3 (TRCN0000065218 and TRCN0000065219), human PI4KB (TRCN0000005692), and mouse ACBD3 (TRCN0000124734 and TRCN0000124738); siRNAs against human ACBD3 (SASI_Hs01_00052488 and SASI_Hs01_00052489), human PI4KB (SASI_Hs01_00149544), human SNAI1 (SASI_Hs01_00039785), and control siRNA (SIC002) from Sigma.

We purchased primary antibodies against ACBD3 (sc-101277) from Santa Cruz; GM130 (#560066) from BD Transduction Laboratories; α-tubulin (#T9026) from Sigma; Flag tag (F3165) and EGFP (G6539) from Sigma; SNAI1 (#3879), PARP1 (#9542), and β-actin (#4970) from Cell Signaling; ZEB1 (21544-1-AP), STC2 (10314-1-AP), and CLU (12289-1-AP) from Proteintech; CDH1 (13–1700) from ThermoFisher Scientific; and VSVG (IE9F9) from Kerafast.

### Cell lines

Human lung cancer cell lines (A549, H1299, CALU1, CALU6, H358, H460, H441, H292, H358, H2122, H1792, and HCC827) and human embryonic kidney 293 T cells were obtained from the American Type Culture Collection. Murine lung cancer cell lines (393 P, 344 P, and 344SQ) were generated as previously described [4]. Human Umbilical Vein Endothelial Cells (HUVECs) were purchased from Lonza. Thy-1 positive cancer-associated fibroblasts (CAFs) were isolated from freshly resected primary human lung adenocarcinomas as previously described [13]. 293 T cells were cultured in Dulbecco’s MEM containing 10% FBS. HUVECs were cultured in EGM-2 Endothelial Cell Growth Medium-2 BulletKit (CC-5035, Lonza). All human and murine lung cancer cells were cultured in RPMI 1640 medium containing 10% FBS. Cells were maintained at 37 °C in a humidified atmosphere with 5% CO2. Cell transfections were carried out using the jetPRIME Versatile DNA/siRNA transfection reagent (Polyplus). Stable cell transfectants were selected using puromycin (for pLKO.1 vectors) or G418 (for pcDNA3.1 and pEGFP-C3 vectors). To generate ACBD3 R223* mutant cells, H441 cells were co-transfected with gRNA (5’-AGAAGAGGAAAGGCTTCGAC-3’), ssDNA Homology-directed repair sequence (5’-TGAAGAGGAAGAAAGAGAACGTCTGCAAAAGGAGGAAGAGAAACGTAGGAGAGAAGAAGAGGAAAGGCTTTGACGCGAGGAAGAGGAAAGGAGACGGATAGAAGAAGAAAGGCTTCGGTTGGAGCAGCAAAAGTAAGTTTATTATG-3’), and Cas9 enzyme (Genscript), according to the manufacturer’s instructions. Single clones were then isolated and verified by genomic DNA sequencing.

### Vector construction

The EGFP-VSV-G (ts045) expression construct (Addgene plasmid #11912) was a gift from Dr. Jennifer Lippincott-Schwartz (Janelia). Human ACBD3-pEGFP and ACBD3 (1–175)-pEGFP were kindly provided by Dr. Carolyn E Machamer (Department of Cell Biology, Johns Hopkins University School of Medicine Baltimore, MD, USA). The mCherry-TOMM20-N-10 construct was a gift from M. Davidson (Addgene plasmid #55146). EGFP-PI4KB was generated as previously described [16]. The human and mouse ACBD3 coding sequences were isolated by performing PCR on EGFP-ACBD3 plasmid and cDNA from 344SQ cells, respectively, and then cloned into pcDNA3.1(-) (Invitrogen) or pEGFP-MAO vector [15]. To generate the ACBD3-MAO fusion proteins, we amplified the C-terminal transmembrane domain sequence (amino acids 479 to 528) of monoamine oxidase A and inserted it downstream of a WT or truncated ACBD3 cassette, as previously described [15]. Truncations and mutations were generated by PCR method. Primers are listed in Table S3.

### Cell proliferation, colony formation, apoptosis, migration, and invasion assays

Cell proliferation assays were conducted using Cell Proliferation Reagent WST-1 (Roche) following the manufacturer’s instructions. Colony formation assays at low density on plastic were performed as previously described [16]. Apoptotic cels were quantified using the eBioscience™ Annexin V Apoptosis Detection Kit (Invitrogen). Migration, invasion, and HUVEC and CAF recruitment assays were performed using uncoated and Matrigel-coated Boyden chambers, respectively, as previously described [12].

### WB analysis and immunoprecipitation assays

WB analysis was performed as previously described [13]. For immunoprecipitation, H1299 cells were transfected with the indicated expression vectors, lysed after 48 h in 1× radioimmunoprecipitation assay (RIPA) buffer (Cell Signaling), and incubated with antibodies at 4 °C overnight. The immune complex was captured with protein G agarose beads (Cell Signaling), washed with 1× RIPA buffer once and 1× PBS three times, and boiled in 1× sodium dodecyl sulfate loading buffer at 98 °C for 10 min. The resulting samples were subjected to WB analysis.

### NOTCH activity assay

NOTCH activity was assessed using the Notch1 Pathway Reporter Kit (#79503, BPS Bioscience) following the manufacturer’s instructions. Briefly, the CBF1/RBP-Jk (CSL) luciferase reporter vector was co-transfected with a Renilla luciferase vector, along with shACBD3, ACBD3 overexpression, or control vectors into H441 or H1299 cells. Luciferase activity was then measured using the Dual-Luciferase® Reporter Assay System (Promega).

### qPCR analysis

Reverse transcription was performed with the qScript cDNA SuperMix kit (Quanta Biosciences). mRNA levels were quantified using SYBR® Green Real-Time PCR Master Mixes (Thermo Fisher Scientific) and normalized to ribosomal protein L32 (Rpl32) mRNA. The specific PCR primers used in this study are listed in Table S3.

### CM sample preparation and transfer

Following a previously described protocol [13], CM samples were isolated, filtered using a 0.45-μm filter, and combined with an equal volume of complete growth medium. This mixture was then applied to cells. In cell proliferation and colony formation assays, CM samples were replaced every two days.

### LC-MS analysis

H2122 cells (2 × 10^6^) were seeded in a 10 cm plate. 24 h after seeding, the cells were washed three times with PBS and incubated in 8 ml serum-free medium. The conditioned medium (CM) samples were collected after 16 h, filtered, and concentrated using Amicon Ultra-15 10 K and Ultra-0.5 10 K centrifugal filters. To solubilize the samples, 25 μL of 5% SDS, 50 mM TEAB (pH 7.55) was added. The solution was centrifuged at 17,000 g for 10 min to remove debris. Proteins were reduced by adding 20 mM TCEP (Thermo Fisher Scientific, 77720) and incubated at 65 °C for 30 min. After cooling to room temperature, 1 μL of 0.5 M iodoacetamide was added, and the solution was allowed to react in the dark for 20 min. Then, 2.75 μL of 12% phosphoric acid was added, followed by the addition of 165 μL of binding buffer (90% methanol, 100 mM TEAB, final pH 7.1). The resulting solution was passed through an S-Trap spin column (protifi.com) using a benchtop centrifuge (30-second spin at 4,000 g). The spin column was washed three times with 400 μL of binding buffer. Trypsin was added to the protein mixture at a ratio of 1:25 in 50 mM TEAB (pH 8), and the solution was incubated at 37 °C for 4 h. Peptides were eluted with 80 μL of 50 mM TEAB, followed by 80 μL of 0.2% formic acid, and finally 80 μL of 50% acetonitrile, 0.2% formic acid. The combined peptide solution was dried using a speed vac and then resuspended in an autosampler vial with 2% acetonitrile, 0.1% formic acid, and 97.9% water.

### Quantification of Golgi area and element size and number

As previously described [17], the area occupied by GM130-stained structures was quantified in volume projections using a limiting polygon. The Golgi area was normalized by dividing it by the nucleus area in the same cell (*n* = 30–50 cells per group). Golgi element size and number were determined from maximum intensity projections of deconvolved, thresholded, and watershed-segmented 3D stacks (*n* > 50 cells per group) using particle analysis (ImageJ, https://imagej.nih.gov/ij/).

### FRAP assays

FRAP experiments were conducted as previously described [17, 41]. In brief, prebleach images were acquired and averaged to determine baseline fluorescence intensity. Following laser-induced bleaching, the initial phase of post-bleach images was captured at maximum speed to monitor the rapid recovery phase, representing the diffusion component. Subsequently, 60–90 images were acquired at 1- and 10-second intervals to capture the slower recovery phase, reflecting the binding component. Time-lapse image stacks were aligned via rigid body transformation and corrected for photobleaching using the average intensity of unbleached control cells. Fluorescence recovery was quantified as previously outlined [17].

### Golgi fraction isolation

As previously described [14], we enriched cell lysates for Golgi fractions using the Minute Golgi Apparatus Enrichment Kit (GO-037, Invent Biotechnologies), following the manufacturer’s instructions.

### Secretory vesicle trafficking assays

For VSV-G assays, H23 cells were transiently transfected with EGFP-VSV-G (ts045) and subjected to a temperature shift from permissive (32 °C) to restrictive (40 °C) temperatures for 20 h. Subsequently, the cells were transferred back to the permissive temperature of 32 °C for 1 h in the presence of 100 µg/mL cycloheximide. After fixation, exofacial and total VSV-G were detected in non-permeabilized cells using an anti-VSV-G antibody and by measuring EGFP signal intensity, respectively. The trafficking of VSV-G to the plasma membrane was quantified based on a ratio of fluorescence signal from exofacial (surface) VSV-G to EGFP (total) signal intensity, as previously described [17].

### Microscopy and image analysis

Cells were imaged using an Eclipse Ti inverted microscope with an A1+ confocal scanner (Nikon, Japan), equipped with diode lasers of 405, 488, 561, and 640 nm wavelengths, high sensitivity Gallium arsenide phosphide and photomultiplier tube detectors, and either a 60 × 1.4 NA Oil or 100 × 1.45 NA Oil objective. NIS-Elements software (Nikon) version 4.40 (Build 1084) was utilized for image acquisition. For high-resolution imaging, Z-stacks were acquired sequentially with slow scan speed, using a frame size of 512 × 512 or 1024 × 1024, low pinhole, and optimized detector gain. Nyquist sampling criteria were followed, and laser power was adjusted to minimize bleaching. Post-acquisition, images were processed and deconvolved using Huygens Professional version 18.04 (Scientific Volume Imaging, The Netherlands) with the Classic Maximum Likelihood Estimation algorithm. Image analysis was performed using Fiji (ImageJ version 1.51 s, NIH), Huygens Professional, or NIS-Elements. Immunofluorescence procedures were carried out following previously described [16]. Radial profile analysis was performed as previously described [42].

### Statistical analysis

Unless stated otherwise, the results shown are representative of replicated experiments and are the means ± standard deviations from triplicate samples or randomly chosen cells within a field. The investigators were not blinded to group allocation during the experiment or outcome assessment. When conducting the correlation analysis and comparing mRNA levels with EMT scores in human lung cancers, the EMT score was calculated as previously described [43]. Boxplots represent 5% (lower whisker), 25% (lower box), 50% (median), 75% (upper box), and 95% (upper whisker). Statistical evaluations were carried out with Prism 6 (GraphPad Software, Inc.). Unpaired 2-tailed Student t-tests were used to compare the mean values of 2 groups. ANOVA with Dunnett’s test was used for comparing multiple treatments to a control. *P*-values < 0.05 were considered statistically significant. Kaplan-Meier survival data were generated using KMPlot (https://kmplot.com) [44]. Plots were generated for the respective groups using Graphpad Prism 6.

Based on our previous studies [12–17], a sample size of *n* ≥ 3 is sufficient to detect significant differences with 80% power for a two-tailed t-test. Similarly, for a one-way ANOVA test, *n* ≥ 3 is adequate to achieve 80% power for detecting significant differences. For animal studies, our prior experience indicates that a sample size of *n* = 5–10 per group is sufficient to detect significant differences among groups using a one-way ANOVA test. No samples or animals were excluded from the analysis. For the ANOVA test, variance similarity between groups was statistically assessed using Graphpad Prism 6.

## Results

### ACBD3 promotes tumor growth by interacting with PI4KB

**Fig. 1:**
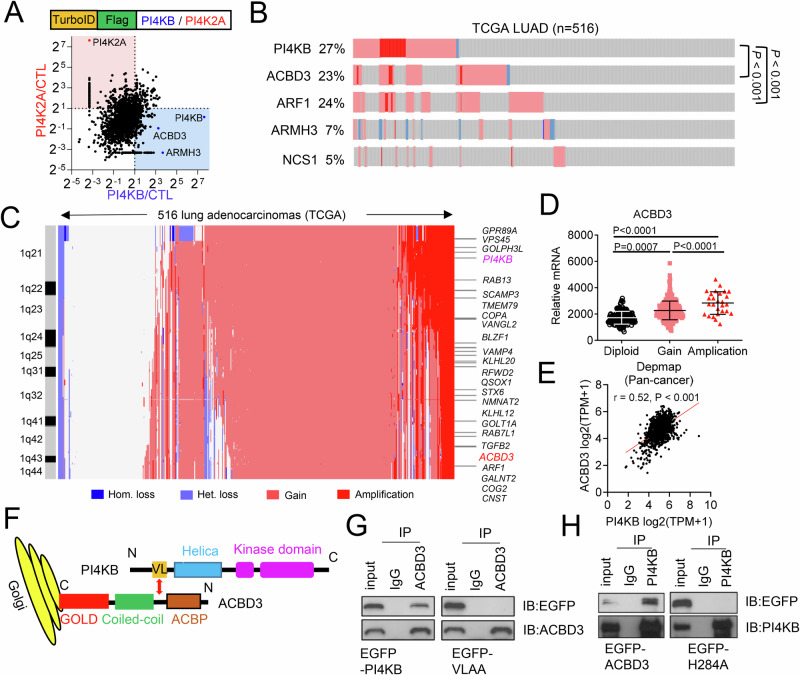
ACBD3 is co-amplified with PI4KB in LUAD. **A** Identification of PI4KB and PI4K2A binding proteins by TurboID assays. x-axis: fold change of protein levels (PI4KB/CTL); y-axis, fold change of protein levels (PI4K2A/CTL). **B** Gene copy number (rows) in TCGA LUAD cohort. P value indicates co-occurrence. **C** Heat map of copy number alterations in Golgi genes (rows) in LUAD (*n* = 516, columns) in The Cancer Genome Atlas (TCGA) cohort. Color codes for copy number changes are indicated. **D** Correlation of ACBD3 mRNA levels and gene copy numbers in TCGA LUAD samples (dots). Diploid (*n* = 143), gain (*n* = 318), and amplification (*n* = 27). **E** PI4KB (x-axis) and ACBD3 (y-axis) mRNA expression in human pan-cancer cell lines (dots) (r and P values, Pearson’s correlation). Data from Depmap (https://depmap.org). **F** Schema: PI4KB is recruited to Golgi by ACBD3. VL: Valine and Leucine amino acids that mediate the interaction between PI4KB and ACBD3. **G** H2122 cells were transfected with GFP-tagged WT or mutant (VLAA) PI4KB and immunoprecipitated with ACBD3 antibody or IgG and the protein level of ACBD3 and GFP-PI4KB were examined by WB. **H** H2122 cells were transfected with GFP-tagged WT or mutant (H284A) ACBD3 and immunoprecipitated with PI4KB antibody or IgG and the protein level of PI4KB and GFP-ACBD3were examined by WB. Data indicate the mean ± SD from a single experiment incorporating biological replicate samples (n = 3, unless otherwise indicated) and are representative of at least 2 independent experiments. P values were determined using Fisher’s exact test (for B) or one-way ANOVA test (for D).

Our recent work has shown that PI4KB is amplified and functions as a cancer driver by heightening the secretion of pro-tumorigenic proteins in chromosome 1q-amplified LUAD (1q-LUAD) [16]. Since PI4KB lacks a Golgi-targeting motif, its Golgi localization is facilitated by other Golgi resident proteins, including ACBD3 [20], ARF1 [45], ARMH3 [46], and NCS1 [47]. To determine which proteins are responsible for recruiting PI4KB in lung cancer cells, we conducted a biotin proximity labeling assay using TurboID-fused PI4KB or PI4K2A as the baits, revealing ACBD3 and ARMH3 as major PI4KB binding partners (Fig. 1A and Table S1). We also investigated the copy number and mRNA level changes of these PI4KB-associated Golgi genes and found that the amplification/overexpression of ACBD3 and ARF1 coincides with PI4KB amplification (Fig. 1B). Thus, ACBD3 may function as the major PI4KB binding protein in 1q-LUAD. ACBD3 and PI4KB are co-located on the same 1q amplicon (Fig. 1C), with ACBD3 mRNA levels positively correlated with its copy number gains/amplifications (Fig.1D). In human cancer cell lines, PI4KB and ACBD3 are co-expressed (Fig. 1E). The interaction between PI4KB and ACBD3 was confirmed by immunoprecipitation (IP)-Western blot (WB) assays, and mutation of the reported binding sites on PI4KB (V55L56) or ACBD3 (H284) abolished their interactions (Fig. 1F–H) [48].

**Fig. 2:**
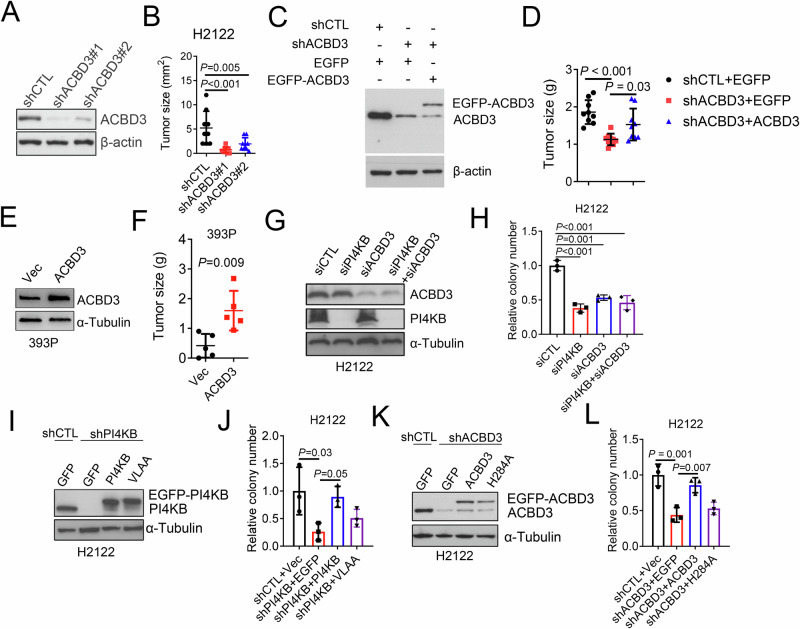
ACBD3 drives NSCLC growth through PI4KB. **A** WB analysis of ACBD3 protein levels in H2122 cells stably transfected with shRNAs against ACBD3 (shACBD3) or control shRNA (shCTL). **B** Othotopic lung tumor size formed by intrathoracically injected H2122 transfectants. *n* = 10. **C** WB analysis of endogenous and ectopic EGFP-tagged ACBD3 (EGFP-ACBD3) protein levels in H2122 cells transfected with shACBD3 or shCTL and EGFP-ACBD3 or EGFP expression vectors. **D** Subcutaneous tumor size formed by cells in (**C**). *n* = 8–10. **E** WB analysis of ACBD3 and EGFP-ACBD3 protein levels in 393 P cells transfected with EGFP-ACBD3 or EGFP expression vectors. **F** Tumor size formed by cells in (**E**). *n* = 5. **G** WB analysis of ACBD3 and PI4KB protein levels in H2122 cells transfected with siRNAs against PI4KB (siPI4KB), ACBD3 (siACBD3), or control siRNA (siCTL). **H** Colony numbers formed soft agar by cells in (**G**). **I** WB analysis of endogenous or EGFP-tagged PI4KB in H2122 cells transfected with shRNAs against PI4KB (shPI4KB) or shCTL and WT or mutant PI4KB (VLAA) expression vectors. **J** Colony numbers in soft agar formed by cells in (**I**). **K** WB analysis of endogenous or EGFP-tagged ACBD3 in H2122 cells transfected with shACBD3 or shCTL and WT or mutant ACBD3 (H284A) expression vectors. **L** Colony numbers in soft agar formed by cells in (**K**). Data indicate the mean ± SD from a single experiment incorporating biological replicate samples (*n* = 3, unless otherwise indicated) and are representative of at least 2 independent experiments. *P*-values were determined using two-tailed Student’s t-test (for **F**) or one-way ANOVA test (for **B**, **D**, **H**, **J**, **L**).

Next, we investigated ACBD3’s role in the growth of 1q-LUAD cells. ACBD3 depletion impaired tumor growth (Fig. 2A, B), which can be restored by ectopic ACBD3 reconstitution (Fig. 2C, D). Conversely, overexpression of ACBD3 in 1q-diploid murine lung cancer 393 P cells increased subcutaneous tumor size (Fig. 2E, F). In support of the hypothesis that ACBD3 and PI4KB act in the same pathway, depletion of ACBD3 or PI4KB similarly reduced 1q-LUAD cell growth (Fig. 2G, H). In PI4KB-deficient cells, reconstitution of wild type (WT) but not the mutant PI4KB that cannot bind to ACBD3 restored colony forming activity (Fig. 2I, J). Similarly, in ACBD3-deficient cells, WT but not the mutant ACBD3 that cannot interact with PI4KB rescued colony forming activity (Fig. 2K, L). Interestingly, ACBD3 depletion impaired the proliferation of 1q-LUAD cells while exhibiting minimal effect on the growth of 1q-diploid lung cancer cells (Fig. S1). These findings suggest that ACBD3 enhances the growth of 1q-LUAD through binding to PI4KB.

### ACBD3 activates pro-tumorigenic secretion

**Fig. 3:**
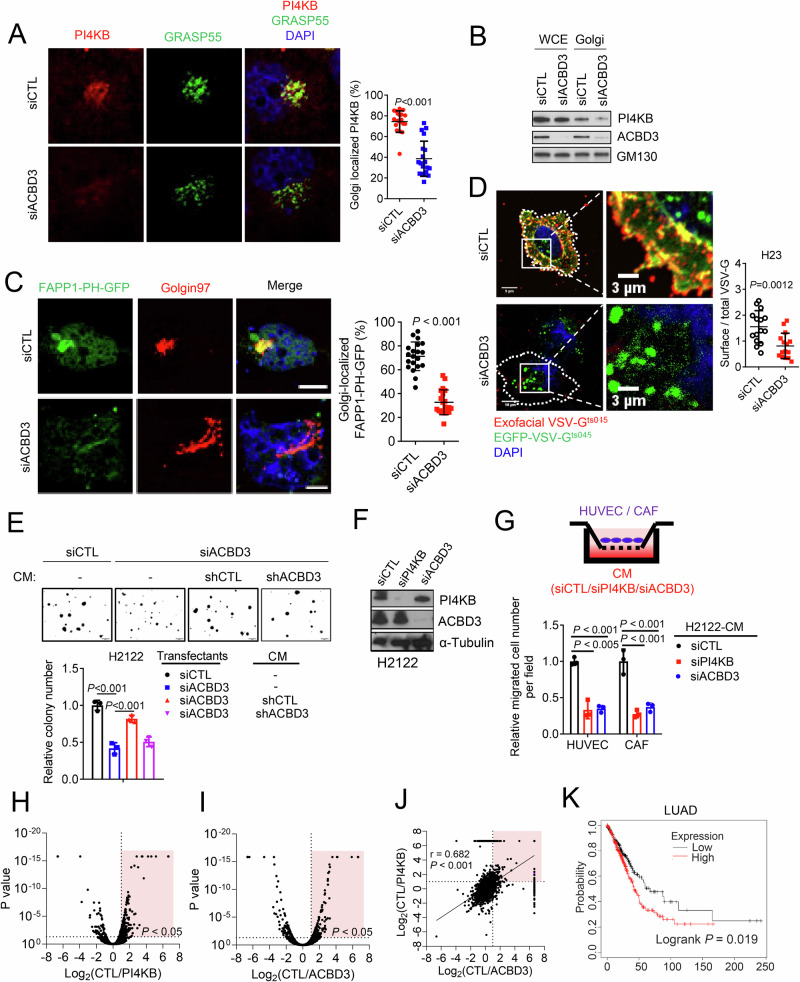
ACBD3 promotes secretion through PI4KB. **A** Confocal micrographs of H23 cells transfected with siCTL or siACBD3 and stained with antibodies against PI4KB (red) and GRASP55 (green). nuclear was stained with DAPI. Golgi localized PI4KB was quantified using ImageJ (plot). *n* = 20. **B** WB analysis of PI4KB and ACBD3 protein levels in whole cell extracts (WCE) and Golgi fractions (Golgi) from H23 cells transfected with indicated siRNAs. **C** Confocal micrographs of H23 cells co-transfected with FAPP-PH-GFP expression vector and siCTL or siACBD3 and stained with Golgin97 antibody. Scale bars:10 μm. Golgi localized FAPP1-PH-GFP levels were quantified (plot). **D** Images of EGFP-VSV-G and siCTL or siACBD3-transfected H23 cells after transfer to the permissive temperature. Total VSV-G was detected by measurement of EGFP signal intensity, and plasma membrane-bound (surface) VSV-G was detected by staining of nonpermeabilized cells with an anti–VSV-G antibody. Scale bars: 3 μm. The scatter plots display the ratio of surface VSV-G to total VSV-G in each cell (dots, *n* = 15 cells per group) for each condition. **E** Colonies formed by shACBD3- and shCTL-transfected H2122 cells treated with conditioned medium (CM) from shCTL- or shACBD3-transfected H2122 cells were imaged and quantified. Scale bar: 200 μm. **F** WB analysis of PI4KB and ACBD3 in H23 cells transfected with indicated siRNAs. **G** Human umbilical vein endothelial cells (HUVEC) and cancer-associated fibroblast (CAF) cells migration in Boyden chambers. CM samples from siRNA-transfected H1299 cells were loaded into the lower wells. **H**, **I** Volcano plots illustration of proteins (dots) identified by LC-MS analysis of CM samples from PI4KB (**H**) or ACBD3 (**I**) deficient H2122 cells. *P*-value (y axis) and fold change (x axis) are shown. Pink quadrant: PI4KB- or ACBD3-controlled secreted proteins. **J** Pearson’s correlation between protein level changes caused by PI4KB and ACBD3 deficiency. Pink quadrant: secreted proteins regulated by both PI4KB and ACBD3. **K** Kaplan-Meier survival analysis of TCGA LUAD cohorts based on gene signatures of secreted proteins. Tumors were scored as being above (high) or below (low) each cohort’s median values. Data indicate the mean ± SD from a single experiment incorporating biological replicate samples (*n* = 3, unless otherwise indicated) and are representative of at least 2 independent experiments. *P*-values were determined using two-tailed Student’s t-test (for **A**, **C**, **D**), one-way ANOVA test (for **E**, **F**), or logrank test (for **K**).

PI4KB-generated Golgi PI4P is indispensable for vesicle biogenesis and oncogenic secretion in 1q-LUAD [16]. Given that ACBD3 interacts with PI4KB, we hypothesize that ACBD3 regulates cancer secretion by modulating the Golgi localization and activity of PI4KB. In agreement with this hypothesis, depletion of ACBD3 led to a decrease in the levels of Golgi-localized PI4KB (Fig. 3A, B) and Golgi PI4P (Fig. 3C), and impaired membrane trafficking of VSV-G (Fig. 3D), an assay used to assess the Golgi secretory activity. In a conditioned medium (CM) transfer experiment, the colony forming activity of ACBD3-deficient cells was partly restored by CM from ACBD3-replete but not -deficient cells (Fig. 3E). Furthermore, CM from ACBD3-deficient cells failed to recruit endothelial and cancer-associated fibroblast cells, a phenotype similar to that observed with PI4KB depletion (Fig. 3F, G), suggesting that ACBD3 functions, at least in part, by regulating secretion.

By profiling the ACBD3- and PI4KB-regulated secretome through liquid chromatography-mass spectrometry (LC-MS) analysis of CM samples, we found that ACBD3 controlled the secretion of proteins similar to those regulated by PI4KB (Fig. 3H–J and Table S2). The high mRNA levels of the secreted proteins predicted shorter survival of LUAD patients (Fig. 3K), implying that they may play oncogenic roles in LUAD progression. By WB, we confirmed that ACBD3 depletion reduced the protein levels of Stanniocalcin-2 (STC2) and Clusterin (CLU), two PI4KB-regulated secretory onco-proteins [16], in the CM samples (Fig. S2A) but not in the cell lysates (Fig. S2B). These findings suggest that ACBD3 modulates pro-tumorigenic secretion through regulating PI4KB.

Our previous work has shown that cancer cells remodel the tumor immune microenvironment (TIME) by modulating the cancer cell secretome [16, 49]. To explore the potential role of ACBD3 in TIME, we examined the correlation between ACBD3 expression and the levels of tumor-infiltrating lymphocytes. We found that ACBD3 expression weakly correlated with the infiltration levels of CD4^+^ T cells (Figure S3), but not with other immune cells, in NSCLC, which argues against the hypothesis that ACBD3-driven secretion modulates TIME.

### ACBD3 maintains Golgi integrity through self-interaction

**Fig. 4:**
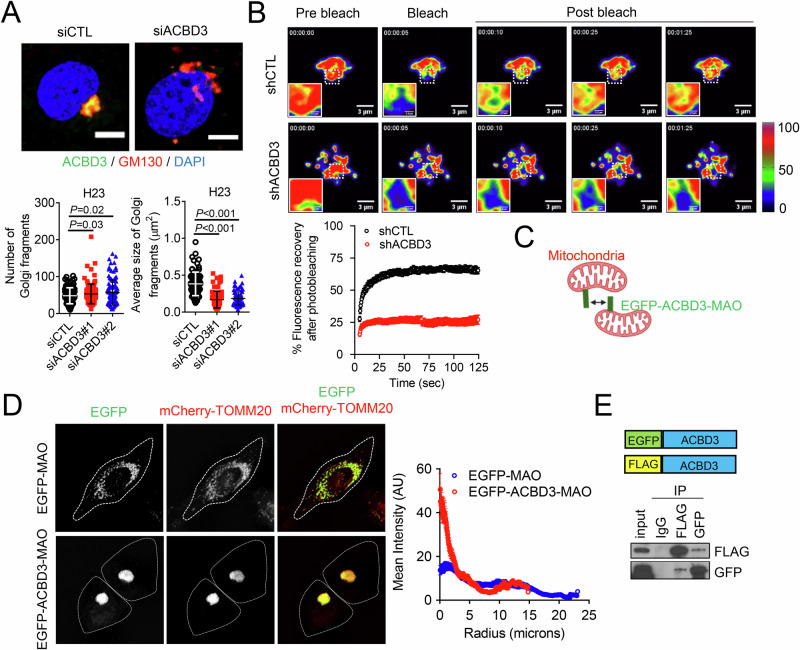
ACBD3 regulates Golgi integrity. **A** Confocal micrographs of Golgi (ACBD3, green; GM130, red) and nuclei (DAPI, magenta) in H23 cells transfected with siACBD3 or siCTL. Scale bars: 5 μm. Scatter plots show the average Golgi element numbers (left) and areas (right). *n* = 80. **B** Pseudocolored images of FRAP assays using the Golgi enzyme GalNAcT in siCTL- (top) and siACBD3- (bottom)transfected H23 cells at time points indicated. Insets: the bleached regions of interest. Intensity levels are indicated by LUT bar (right). Scale bars: 3 μm. The scatter plot shows the intensity recovery profile (%) after photobleaching based on the FRAP assays. *n* = 20. **C** A schema showing that EGFP-ACBD3-monoamine oxidase (MAO) chimeras is localized on the outer membrane of mitochondria. When ACBD3 can form dimers or oligomers, it can cause mitochondria aggregation. **D** Confocal micrographs of H23 cells co-transfected with EGFP-MAO or EGFP-ACBD3-MAO chimeras and mCherry-tagged mitochondria marker TOMM20. Co-localization of EGFP-MAO/EGFP-ACBD3-MAO and TOMM20 is illustrated in the far-right panel. Scale bars: 10 μm. Radial profile plot based on EGFP signal. *n* = 10. **E** Schema: EGFP- and Flag-tagged ACBD3 expression vectors. H23 cells were co-transfected with EGFP- and FLAG-ACBD3 constructs and immunoprecipitated with FLAG or GFP antibodies or IgG. GFP- and Flag-ACBD3 protein levels in the immunoprecipitants were examined by WB analysis. Data indicate the mean ± SD from a single experiment incorporating biological replicate samples and are representative of at least 2 independent experiments. P values were determined using one-way ANOVA test (for A).

The Golgi apparatus consists of a series of stacked cisternae that are laterally connected by membranous tubules [50]. Golgi integrity is crucial for the sorting, processing, and packaging of proteins into secretory vesicles that bud off from the Golgi and move to their intended destinations [51]. Because ACBD3 is mainly expressed in the Golgi, we examined its role in Golgi structure maintenance. Depletion of ACBD3 led to Golgi dispersal, characterized by increased number and decreased size of Golgi elements (Fig. 4A and Fig. S4A). To determine whether ACBD3 regulates Golgi ribbon linking and intra-organellar connectivity, we performed fluorescence recovery after photobleaching assays using a GFP-tagged Golgi enzyme, N-acetylgalactosaminyltransferase, as previously reported [17], which showed that recovery was significantly slower in ACBD3-depleted cells (Fig. 4B). We speculate that ACBD3 may self-interact to enhance Golgi integrity, similar to Golgi reassembly-stacking protein of 55 kDa (GRASP55) [50]. To test this point, we fused EGFP-tagged ACBD3 (EGFP-ACBD3) with the carboxyl-terminus of Monoamine oxidase (MAO) [42] to anchor the fusion protein to the outer membrane of mitochondria. Interestingly, the expression of EGFP-ACBD3, but not EGFP, led to mitochondrial aggregation (Fig. 4C, D). In a serial deletion assay, we found that the N-terminal (1-78 aa) is not required for ACBD3 self-interaction, while the other domains are sufficient to cause mitochondrial aggregation (Fig. S4B and C). In a co-IP assay, EGFP- and Flag-tagged ACBD3 can pull down each other (Fig. 4E). These findings suggest that ACBD3 maintains Golgi integrity through its self-interaction.

### ACBD3 negatively regulates lung cancer metastasis in 1q-diploid NSCLC

**Fig. 5:**
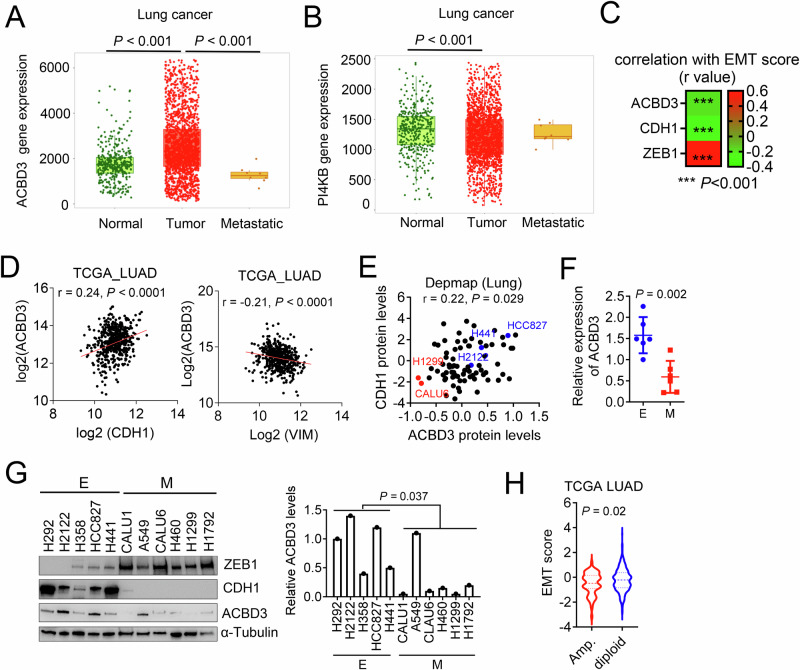
ACBD3 is increased in primary tumors but decreased in metastatic mesenchymal cells. **A**, **B** ACBD3 (**A**) and PI4KB (**B**) mRNA levels of in normal lung tissue (*n* = 391), lung tumor (*n* = 1865), and metastasis (*n* = 8). Data from TNMplot (https://tnmplot.com). **C** Correlation between ACBD3, CDH1, or ZEB1 mRNA levels and EMT scores. Higher EMT score indicated more mesenchymal status. **D** Pearson’s correlation between ACBD3 mRNA levels and CDH1 (left) or ZEB1 (right) in the TCGA LUAD dataset. *n* = 191. **E** Pearson’s correlation between ACBD3 and CDH1 protein levels in human lung cancer cell lines. *n* = 76. **F**, **G** qPCR (**F**) and WB analysis (**G**) of ACBD3 mRNA and protein levels in human NSCLC cell lines classified as epithelial (E) or mesenchymal (M), respectively. ACBD3 protein levels in the cell lines were quantified and normalized to H292 cell line. *n* = 6. **H** EMT scores of LUAD with 1q-amplifications (Amp., *n* = 63) or without amplifications (diploid, *n* = 452). Data indicate the mean ± SD from a single experiment incorporating biological replicate samples and are representative of at least 2 independent experiments. *P*-values were determined using two-tailed Student’s t-test (for **F**–**H**), one-way ANOVA test (for **A**, **B**), or Pearson’s correlation (for **C**, **E**).

Consistent with its role in promoting tumor growth, ACBD3 expression levels are higher in human lung cancers compared to normal tissues (Fig. 5A). However, ACBD3 expression is decreased in the metastatic lesions of several cancer types, including NSCLC (Fig. 5A and Fig. S5A and B), a trend not observed in PI4KB expression levels (Fig. 5B). This aligns with a previous research showing that ACBD3 is among the most downregulated genes in small cell lung cancer, a highly metastatic lung cancer subtype, compared to NSCLC [52], implying that ACBD3 may act as a negative regulator of lung cancer metastasis.

EMT plays a critical role in the initiation of cancer metastatic dissemination [53]. Notably, ACBD3 expression levels exhibit a negative correlation with EMT scores [43] in the TCGA LUAD cohort (Fig. 5C). The expression levels of ACBD3 are positively correlated with epithelial marker E-Cadherin (CDH1) and negatively correlated with mesenchymal marker Vimentin (VIM) or Zinc Finger E-Box Binding Homeobox 1 (ZEB1) in TCGA LUAD cohort (Fig. 5D) and cancer cell lines (Fig. 5E and Fig. S5C and D). In a panel of human lung cancer cell lines, both ACBD3 mRNA and protein levels are downregulated in mesenchymal-like cells (Fig. 5F, G). Interestingly, LUAD with 1q amplifications exhibit a more epithelial phenotype, as indicated by a lower EMT score (Fig. 5H).

**Fig. 6:**
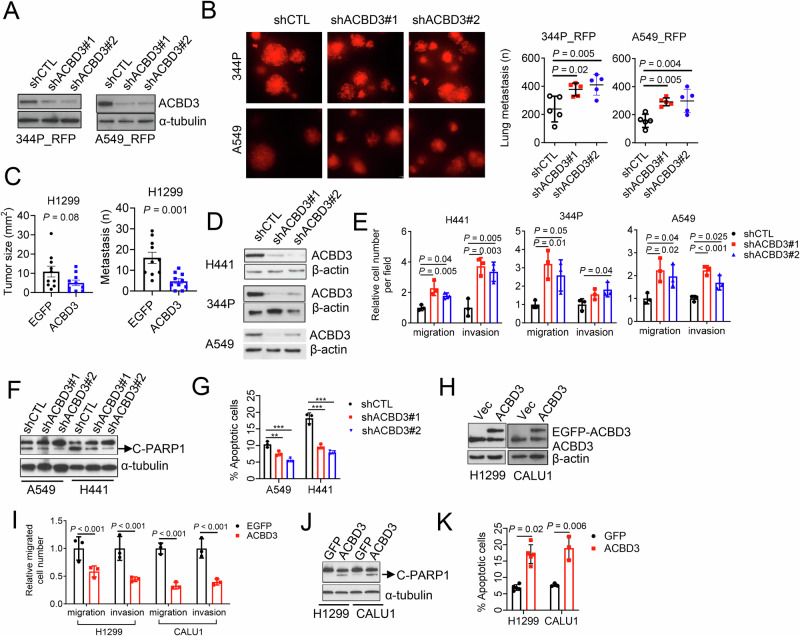
ACBD3 negatively regulates metastasis. **A** WB analysis of ACBD3 protein levels in red fluorescent protein (RFP)-labeled A549 and 344P cells transfected with indicated shRNAs. **B** Lung metastases in syngeneic, immunocompetent mice (344SQ) or nude mice (A549) injected by tail vein with RFP–tagged 344P or A549 cells, respectively. Numbers of metastasis were quantified under fluorescence microscope. *n* = 5. **C** Primary lung tumor size (left) and number of metastasis (right) generated by orthotopically injected H1299 transfectants. *n* = 10. **D** WB analysis of ACBD3 protein levels in H441, 344 P, and A549 cells stably transfected with indicated shRNAs. **E** Boyden chamber transwell migration and invasion assays on H441 (left), 344 P (middle), and A549 (right) transfectants. **F** WB analysis of cleaved PARP1 (C-PARP1) in A549 and H441 transfectants under suspension conditions. **G** Annexin V/propidium iodide flow cytometric analysis of the apoptotic fractions in cells in (**F**). **H** WB analysis of ACBD3 and EGFP-ACBD3 protein levels in H1299 and CALU1 transfectants. **I** Boyden chamber transwell migration and invasion assays on cells in (**H**). **J** WB analysis of C-PARP1 in H1299 and CALU1 transfectants under suspension conditions. **K** Annexin V/propidium iodide flow cytometric analysis of the apoptotic fractions in cells in (**J**). Data indicate the mean ± SD from a single experiment incorporating biological replicate samples (*n* = 3, unless otherwise indicated) and are representative of at least 2 independent experiments. *P*-values were determined using two-tailed Student’s t-test (for **C**, **I**, **K**), one-way ANOVA test (for **B**, **E**, **G**).

Next, we investigated the role of ACBD3 in lung cancer metastasis. Depletion of ACBD3 enhanced the metastatic potential of 1q-diploid NSCLC cells, as demonstrated by the increased number of lung metastases following intravenous injection of ACBD3-depleted A549 and 344 P cells (Fig. 6A, B). Conversely, the metastatic capacity of orthotopic H1299 lung tumors were dampened by overexpression of ACBD3 (Fig. 6C). Enhanced cell motility and anoikis resistance promote the metastatic dissemination of cancer cells [15, 54]. In ACBD3 knockdown epithelial NSCLC cells, we observed a slight decrease in cell proliferation but a significant increase in cell migration and invasion in Boyden chamber transwell assays (Fig. 6D, E; Fig. S6A and B). Moreover, anoikis was inhibited in ACBD3-defient cells (Fig. 6F, G). Conversely, ectopic expression of ACBD3 in mesenchymal NSCLC H1299 and CALU1 cells suppressed cell migration and invasion and enhanced anoikis without affecting cell proliferation on monolayer (Fig.6H–K; Fig. S6C and D), suggesting that ACBD3 acts as a suppressor of NSCLC metastasis.

**Fig. 7:**
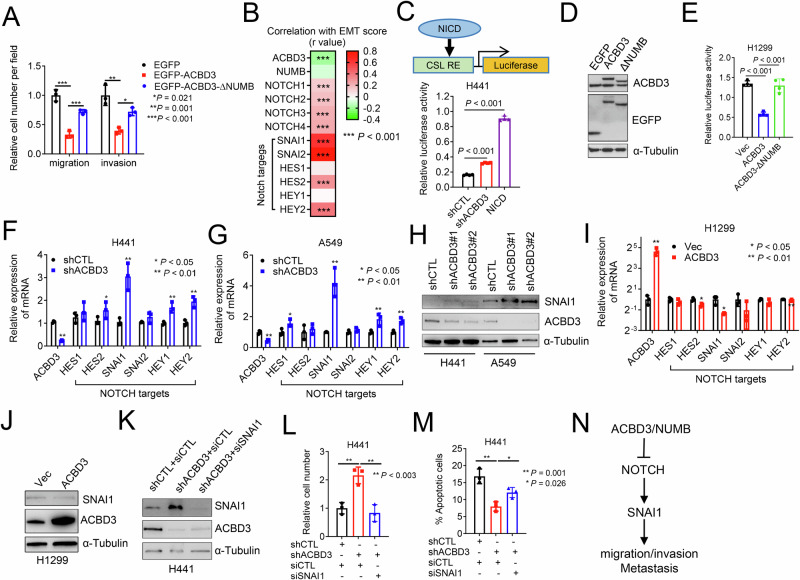
ACBD3 suppresses cancer cell motility and anoikis resistance by inhibiting the NOTCH-SNAI1 axis. **A** Boyden chamber transwell migration and invasion assays in H1299 cells transfected with EGFP-ACBD3 or EGFP-ACBD3 lacking the NUMB-binding domain (ΔNUMB). **B** Correlation between ACBD3, NUMB, NOTCHs, or NOTCH target genes mRNA levels and EMT scores. **C** Schema: The NOTCH luciferase reporter. CBF1/RBPJκ/Suppressor of Hairless/Lag-1 (CSL) response element (RE) is fused upstream of the firefly luciferase gene. NICD Notch intracellular domain, Graph luciferase reporter assay in H441 cells co-transfected with luciferase reporter and shACBD3 or NOTCH1 NICD. *n* = 4. **D** WB analysis of ACBD3 and EGFP-ACBD3 proteins in H1299 cells transfectants. **E** NOTCH luciferase reporter assay. **F**, **G**, **I** qPCR analysis of mRNA levels of ACBD3 and NOTCH target genes in shACBD3-tranfected H441 (**F**) and A549 (**G**), and ACBD3-transfected H1299 (**I**) cells. *n* = 4. **H**, **J**, **K** WB analysis of SNAI1 and ACBD3 protein levels in lung cancer cells transfected with shACBD3 (**H**), ACBD3 expression vector (**J**), or shACBD3 and SNAI1 siRNA (**K**). **L**, **M** Transwell migration (**L**) and apoptosis (**M**) assays of cell transfectants in (**K**). **N** Working model: ACDB3 inhibits the NOTCH-SNAI1 axis to suppress cancer cell motility and metastasis. Data indicate the mean ± SD from a single experiment incorporating biological replicate samples (*n* = 3, unless otherwise indicated) and are representative of at least 2 independent experiments. *P*-values were determined using two-tailed Student’s t-test (for **F**, **G**, **I**), one-way ANOVA test (for **A**, **E**, **L**, **M**).

To determine whether ACBD3’s anti-migratory role depends on its Golgi localization, we deleted its Golgi-dynamics (GOLD) domain, which is essential for the Golgi localization of ACBD3 [55]. Surprisingly, cytosolic ACBD3 retained its anti-migratory function (Fig. S6E–G). However, further deletion of the central domain abolished this effect (Fig. S6G), suggesting that the region between the Acyl-CoA Binding Protein (ACBP) domain and the GOLD domain, rather than its Golgi localization, is responsible for the anti-migratory effect. A previous study had shown that ACBD3 binds to NUMB through its central domain and works synergistically to suppress NOTCH activity, thereby influencing neural progenitor cell fate [29]. NOTCH signaling plays a critical role in driving cell motility, anoikis resistance, and metastasis [56, 57]. We hypothesized that ACBD3 impedes metastasis by interacting with NUMB and attenuating NOTCH activity. Deletion of ACBD3’s NUMB binding domain [29] partially reduced its ability to suppress cell migration (Fig. 7A and Fig. S7A), implying that ACBD3 may function through regulating the NUMB-NOTCH axis. Expression levels of NOTCHs and their target genes (SNAI1, SNAI2, HES2, HEY2) are positively correlated with EMT scores in LUAD (Fig. 7B), indicating activation of the NOTCH pathway in mesenchymal lung cancer cells. Depletion of ACBD3 in epithelial human lung cancer cells increased NOTCH reporter activity (Fig. 7C) [58]. Conversely, overexpression of ACBD3 in mesenchymal lung cancer cells reduced NOTCH activity, and deletion of the NUMB binding domain abolish this effect (Fig. 7D, E). Depletion of ACBD3 in epithelial NSCLC cells increased the expression of NOTCH targets, including the pro-metastatic gene SNAI1 [59] (Fig. 7F–H). In contrast, ectopic expression of ACBD3 in mesenchymal H1299 cells decreased the expression of SNAI1 (Fig. 7I, J). Knockdown of SNAI1 mitigated ACBD3 deficiency-induced cell migration and invasion and anoikis resistance in epithelial H441 cells (Fig. 7K–M and Fig. S7B), suggesting that ACBD3 suppresses lung cancer cell dissemination through inhibiting the NOTCH-SNAI1 axis (Fig. 7N).

### ACBD3 loss-of-function mutations enhance cell motility

By examining the genetic alterations in the *ACBD3* gene across human cancers, we observed recurring nonsense mutations (R223*) and frameshift mutations (R190Gfs*) (Fig. S8A), in addition to gains in gene copy numbers. These mutations lead to the generation of truncated ACBD3 proteins, which intriguingly localize to the nucleus and exhibit instability (Fig. S8B and C). The R223* mutation exposes three classical nuclear localization signals (NLS) within the middle of the ACBD3 protein, while the R190Gfs* mutation generates four de novo NLS (Fig. S8D). Supporting the role of NLS motifs in nuclear translocation, a truncated ACBD3 protein (1-175 aa) lacking the NLS sequence is expressed in the cytosol and remains stable in H1299 cells (Fig. S8B and C). These findings suggest that these recurring mutations may result in reduced ACBD3 protein levels. In the highly metastatic ovarian cancer cell line TOV21-G, which harbors a heterogeneous R190fs* mutation, ectopic expression of ACBD3 suppressed both cell migration and invasion (Fig. S8E and F). Conversely, introducing a R223* mutation in *ACBD3* WT H441 cells via the CRISPR/Cas9 method reduced ACBD3 protein levels and enhanced migratory activity and anoikis resistance (Fig. S8G–I). These findings offer additional evidence that the loss of ACBD3 promotes cancer metastasis.

## Discussion

Cancer development is a complex process involving various stages characterized by mutations and selective pressures that drive cells towards increased proliferation, survival, migration/invasion, and metastasis. Cancer cells demonstrate the ability to transition between different states, including dormant, rapidly proliferating, and metastatic states, through genetic and epigenetic alterations. In this study, we demonstrated that ACBD3 promotes the growth of primary lung tumors with 1q amplifications but acts as a suppressor of metastasis in 1q-diploid lung cancer cells. This represents a novel example of the dichotomous role of a Golgi gene in human cancer progression. Understanding the dichotomous nature of gene function in cancer has significant implications for cancer diagnosis, prognosis, and treatment strategies. Genes exhibiting such dichotomy, including TGF-β1 [60], AMPK [61], and ACBD3, may serve as valuable biomarkers for assessing cancer status and stage. Due to the heterogeneous nature of human cancers, targeting genes with dichotomous roles for therapeutic intervention requires caution, as their inhibition may suppress certain cancer cell subtypes while promoting others. For instance, lung cancer patients with 1q-amplified LUAD, which express high levels of ACBD3, may benefit from therapies targeting ACBD3. However, in 1q-diploid tumors, ACBD3 inhibition could promote metastatic dissemination. Therefore, a comprehensive understanding of the function of potential cancer targets and thorough genetic profiling of tumor tissues are crucial for developing effective and personalized cancer therapies.

A recent study demonstrated that 1q-amplified cancer cells are particularly dependent on this alteration for malignant growth [62], suggesting that genes encoded by the 1q amplicon are pro-tumorigenic. This finding is consistent with our previous work, which showed that amplified PI4KB in the 1q amplicon promotes oncogenic secretion, fueling tumor growth [16]. Notably, 1q-amplified lung tumors exhibit a more epithelial phenotype compared to 1q-diploid tumors (Fig. 5H), implying that an epithelial status may be advantageous for primary tumor growth. The “go-or-grow” hypothesis suggests that cancer cells can reversibly switch between migratory and proliferative states depending on the context [63]. EMT, which drives cancer cells into a motile state, and mesenchymal-epithelial transition (MET), which switches cells back to a proliferative state, are closely linked to the “go-or-grow” concept. In this study, we found that the growth-promoting Golgi protein ACBD3 is amplified and overexpressed in epithelial 1q-LUAD cells but downregulated in migratory mesenchymal cancer cells, further supporting the dichotomous relationship between tumor growth and dissemination. We previously reported a shift from PI4KB to PI4K2A dependency during epithelial-mesenchymal transition (EMT). Epithelial lung cancer cells rely more on PI4KB for Golgi PI4P generation, whereas mesenchymal cells predominantly utilize PI4K2A [12]. In epithelial cancer cells with impaired NOTCH activity, ACBD3 primarily activates the PI4KB-driven secretory pathway to promote tumor growth, particularly in 1q-LUAD cells. In contrast, mesenchymal lung cancer cells are less reliant on the PI4KB pathway but highly dependent on NOTCH signaling for metastatic dissemination. In this context, ACBD3 has minimal or moderate impact on cell proliferation but acts as a negative regulator of NOTCH signaling, resulting in reduced SNAI1 expression and impaired cell motility in mesenchymal lung cancer cells.

Interestingly, analysis of the TCGA dataset revealed recurrent frameshift and nonsense mutations in the ACBD3 gene, leading to the production of truncated ACBD3 proteins that are translocated into the nucleus and subsequently degraded. Notably, all these mutations are heterozygous. Homozygous R190G*fs or R223* mutations will eliminate WT ACBD3 proteins, which may not be beneficial for cancer cells. How these truncated proteins are degraded within the nucleus and whether they have functional roles remain open questions. Nevertheless, these mutations result in a reduction in ACBD3 protein levels. Given ACBD3’s migration-suppressive role in 1q-diploid cancer cells, such mutations may drive the metastatic dissemination of cancer cells. Therefore, *ACBD3* gene is either amplified in certain cancer cells to promote tumor growth or mutated in others to enhance metastasis.

This study has several limitations. First, while we have previously confirmed the autocrine roles of STC2 and CLU in 1q-amplified LUAD cells [16], we have not yet identified the specific secreted proteins that may function in a paracrine manner to recruit HUVEC and CAF cells. Second, mesenchymal NSCLC cells express lower levels of ACBD3 but exhibit a compact Golgi structure [17], which appears contradictory given ACBD3’s critical role in maintaining Golgi integrity. A possible explanation is that other Golgi proteins, such as PAQR11 [17] and PI4K2A [12], which are highly expressed in mesenchymal cancer cells, may compensate for the changes in Golgi morphology caused by ACBD3 loss. Further investigation is required to test this hypothesis. Third, we have yet to determine which NOTCH mediates ACBD3’s regulation on SNAI1. NOTCH1, NOTCH2, and NOTCH4 have all been reported to transactivate SNAI1 [58, 64], and additional work is needed to identify the NOTCH mediator in 1q-diploid NSCLC cells.

## Supplementary information

Supplementary figures and legends

Supplementary table 3

Supplementary table 1

Supplementary table 2

## Data Availability

All data associated with this study are present in the paper or in the Supplementary Materials.
