# Supplementary material for: Dichotomous roles of ACBD3 in NSCLC growth and metastasis
Source: Oncogene. 2025 Apr 6;44(25):2078–90. doi: 10.1038/s41388-025-03360-w (PMC12167707; doi:10.1038/s41388-025-03360-w)
Supplement: Supplementary file 1 — Supplementary figures and legends [file 41388_2025_3360_MOESM1_ESM.pdf]

Supplementary figures

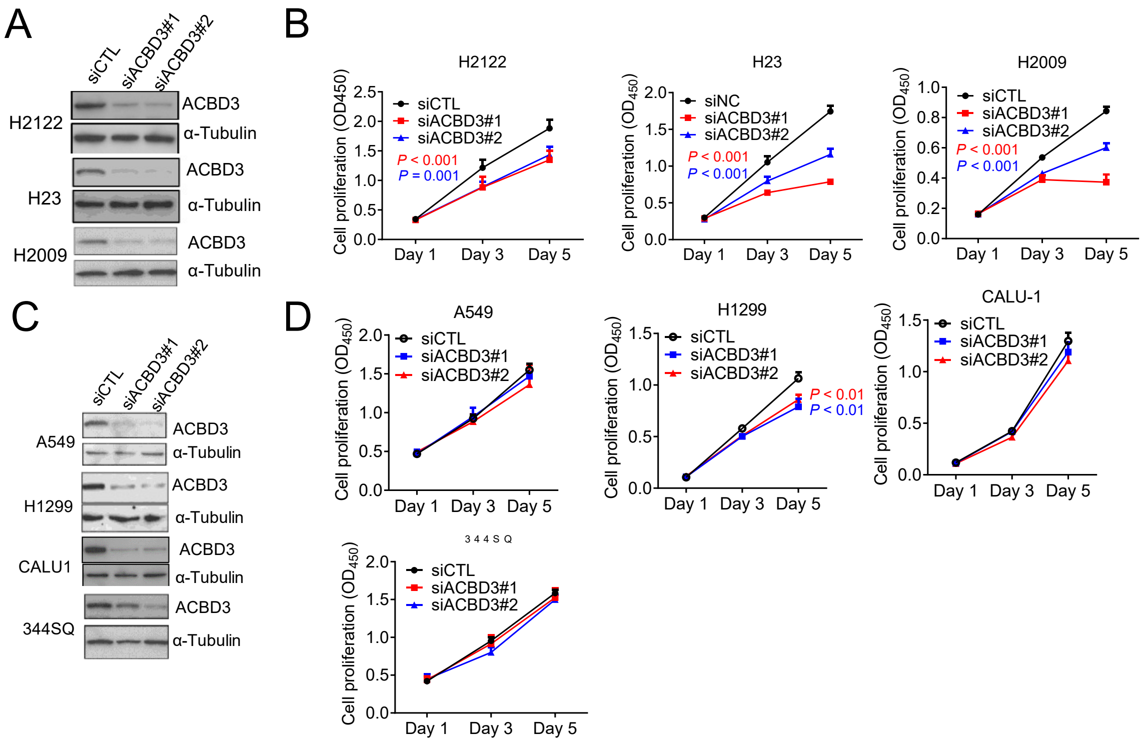

Figure S1. ACBD3 drives 1q-NSCLC growth. (A and C) WB analysis of ACBD3 protein levels in 1q-amplified (A) and diploid (C) NSCLC cells transfected with siACBD3 or siCTL. (B and D) WST-1 cell proliferation assay on 1q-amplified (B) and diploid (D) NSCLC cells. Data indicate the mean ± SD from a single experiment incorporating biological replicate samples (n = 3, unless otherwise indicated) and are representative of at least 2 independent experiments. P values were determined using one-way ANOVA test.

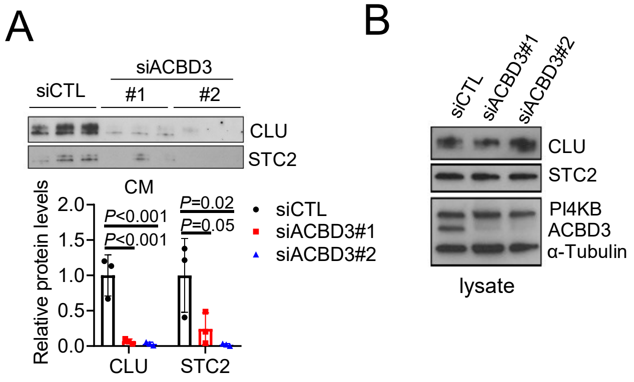

Figure S2. ACBD3 regulates oncogenic secretion. (A and B) WB analysis of CLU, STC2, PI4KB, and ACBD3 protein levels in CM (A) and whole cell lysate (B) from H2122 cells transfected with siCTL or siACBD3. Data indicate the mean ± SD from a single experiment incorporating biological

replicate samples (n = 3, unless otherwise indicated) and are representative of at least 2 independent experiments. P values were determined using one-way ANOVA test.

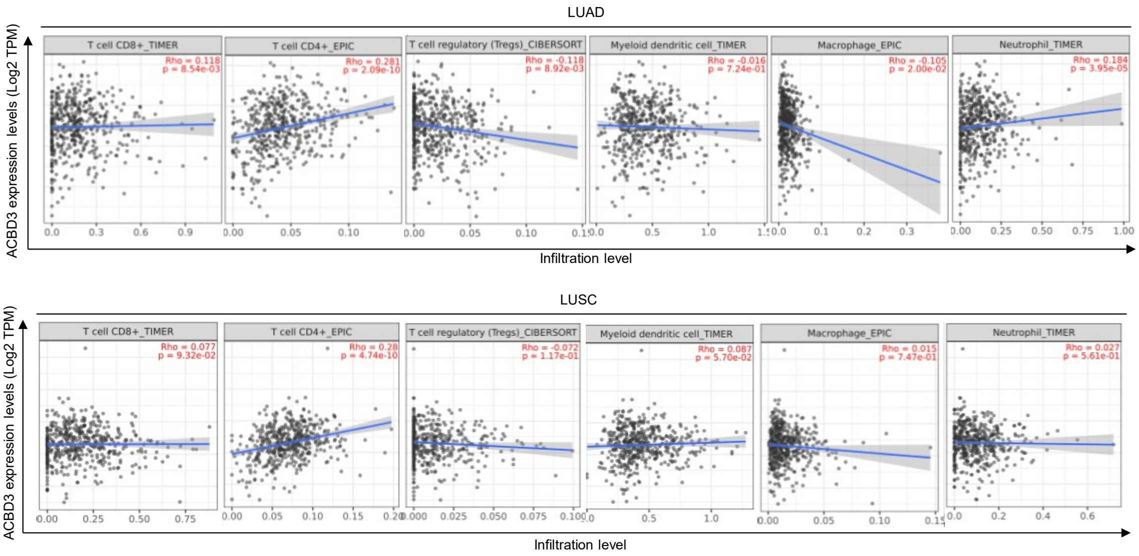

Figure S3. Correlation between ACBD3 expression and tumor-infiltrating lymphocyte levels. Data obtained from TIMER2.0 (http://timer.cistrome.org/).

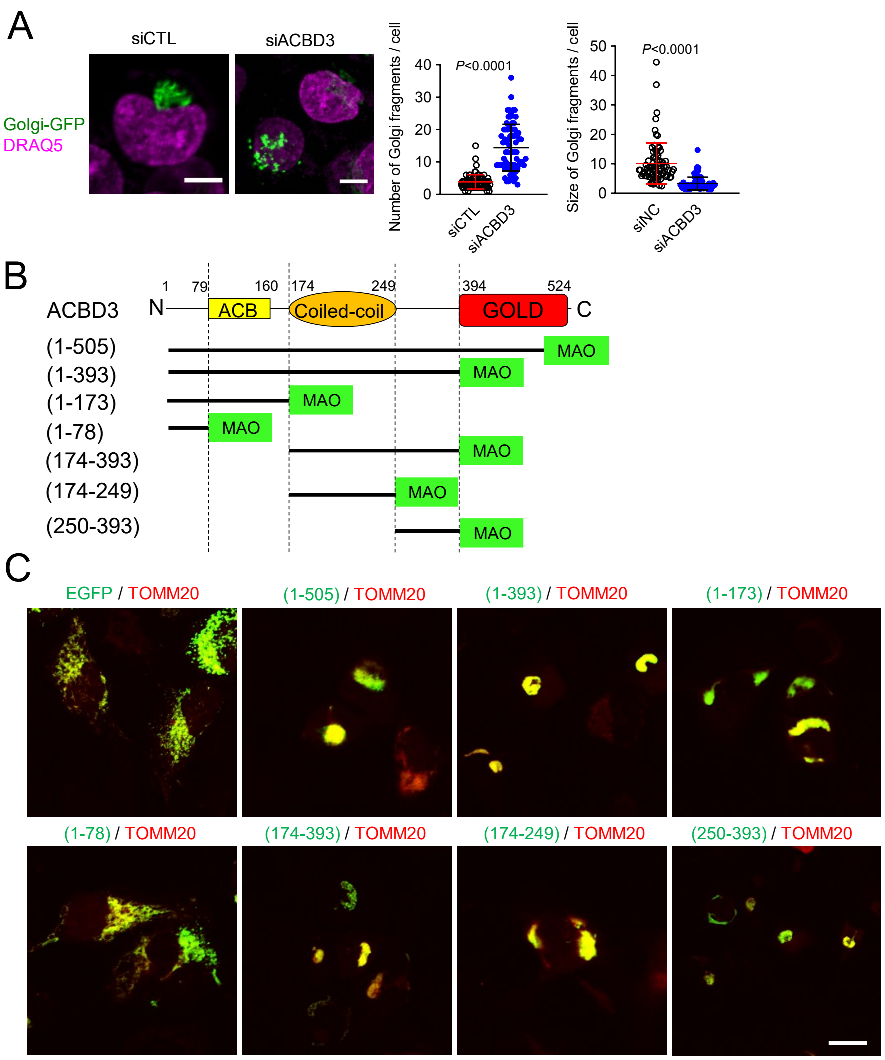

Figure S4. ACBD3 regulates Golgi compaction. (A) Confocal micrographs of Golgi (ACBD3, green; GM130, red) and nuclei (DAPI, magenta) in A549 cells transfected with siACBD3 or siCTL. Scale bars: 5 μm. Scatter plots show average Golgi element numbers (left) and areas (right). (B) EGFP-ACBD3-MAO chimeras constructs. (C) Confocal micrographs of H23 cells co-transfected with EGFP-MAO or EGFP-ACBD3-MAO chimeras constructs in (B) and mCherry-tagged mitochondria marker TOMM20. Scale bars: 10 μm. Data indicate the mean ± SD from a single experiment incorporating biological replicate samples (n = 3, unless otherwise indicated) and are representative of at least 2 independent experiments. P values were determined using two-tailed Student's t-test.

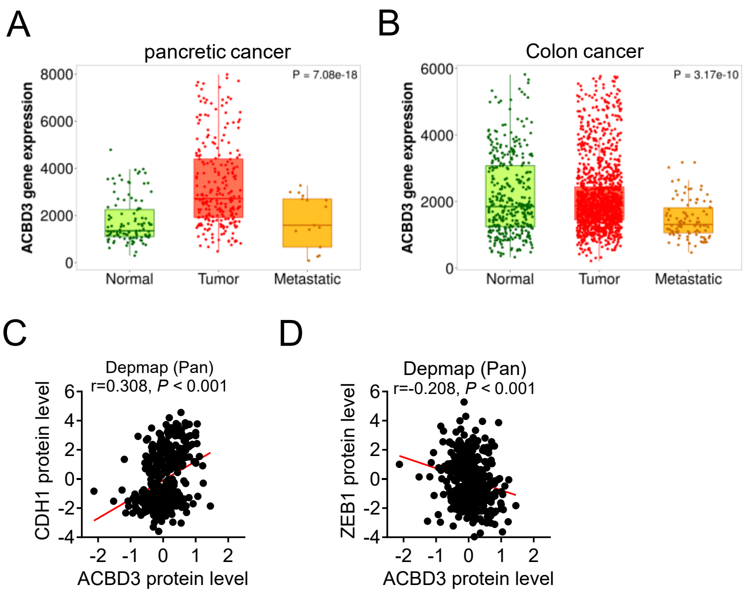

Figure S5. ACBD3 expression levels are negative correlated with EMT. (A) ACBD3 mRNA levels in normal tissue (n = 108), tumor (n = 248), and metastasis (n = 17) from pancreatic cancer patients. (B) ACBD3 mRNA levels in normal tissue (n = 143), tumor (n = 1450), and metastasis (n = 99) from colon patients. (C and D) Correlation between ACBD3 and CDH1 (C) or ZEB1 (D) protein levels in pan-cancer cell lines (Depmap). Data indicate the mean ± SD from a single experiment incorporating biological replicate samples (n = 3, unless otherwise indicated) and are representative of at least 2 independent experiments. P values were determined using one-way ANOVA test (for A and B) or Pearson’s correlation analysis (for C and D).

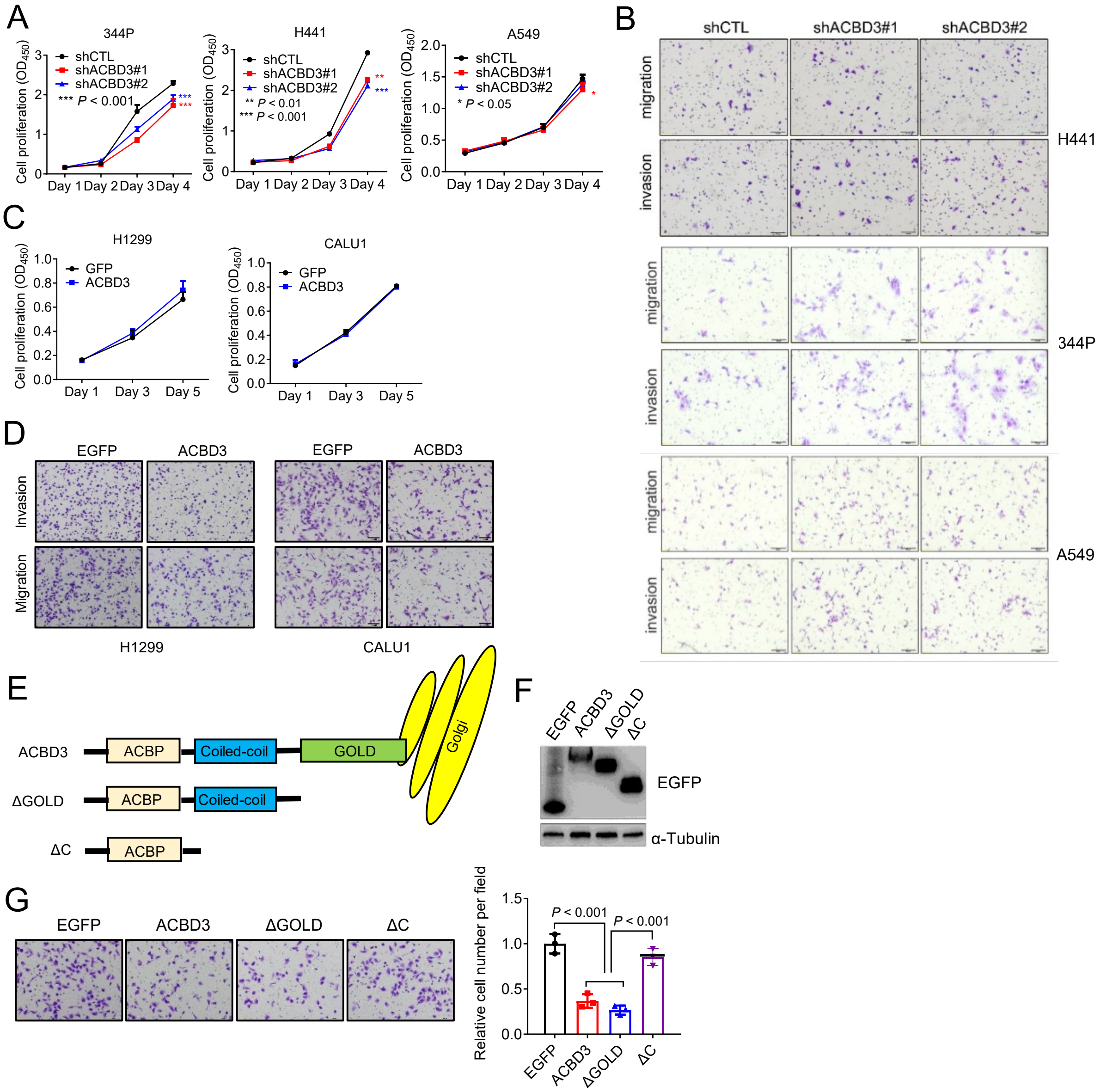

Figure S6. ACBD3 negatively regulates cell migration and invasion. (A) WST-1 cell proliferation assay in NSCLC cells transfected with ACBD3 shRNAs (shACBD3) or control shRNA (shCTL). (B) Representative images of migrated/invaded H441, 344P, and A549 transfectants in Boyden chambers. (C) WST-1 cell proliferation assay in mesenchymal NSCLC cells transfected with either an ACBD3 expression vector or an EGFP control vector. (D) Representative images of migrated/invaded H1299 and CALU1 transfectants in Boyden chambers. (E) Schema of truncated ACBD3 expression constructs. (F) WB analysis of EGFP-fused ACBD3 proteins in H1299 transfectants. (G) Boyden chamber transwell migration assay of the cells described in (F), with migrated cells imaged and quantified (graph). Data indicate the mean ± SD from a single experiment incorporating biological replicate samples (n = 3, unless otherwise indicated) and are representative of at least 2 independent experiments. P values were determined using two-tailed Student's t-test (for C) or one-way ANOVA test (A and G).

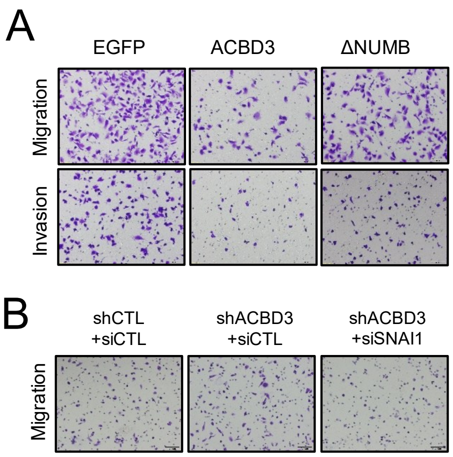

Figure S7. ACBD3 suppresses cell motility by inhibiting the NOTCH-SNAI1 axis. (A and B) Representative images of migrated/invaded H1299 (A) and H441 (B) transfectants.

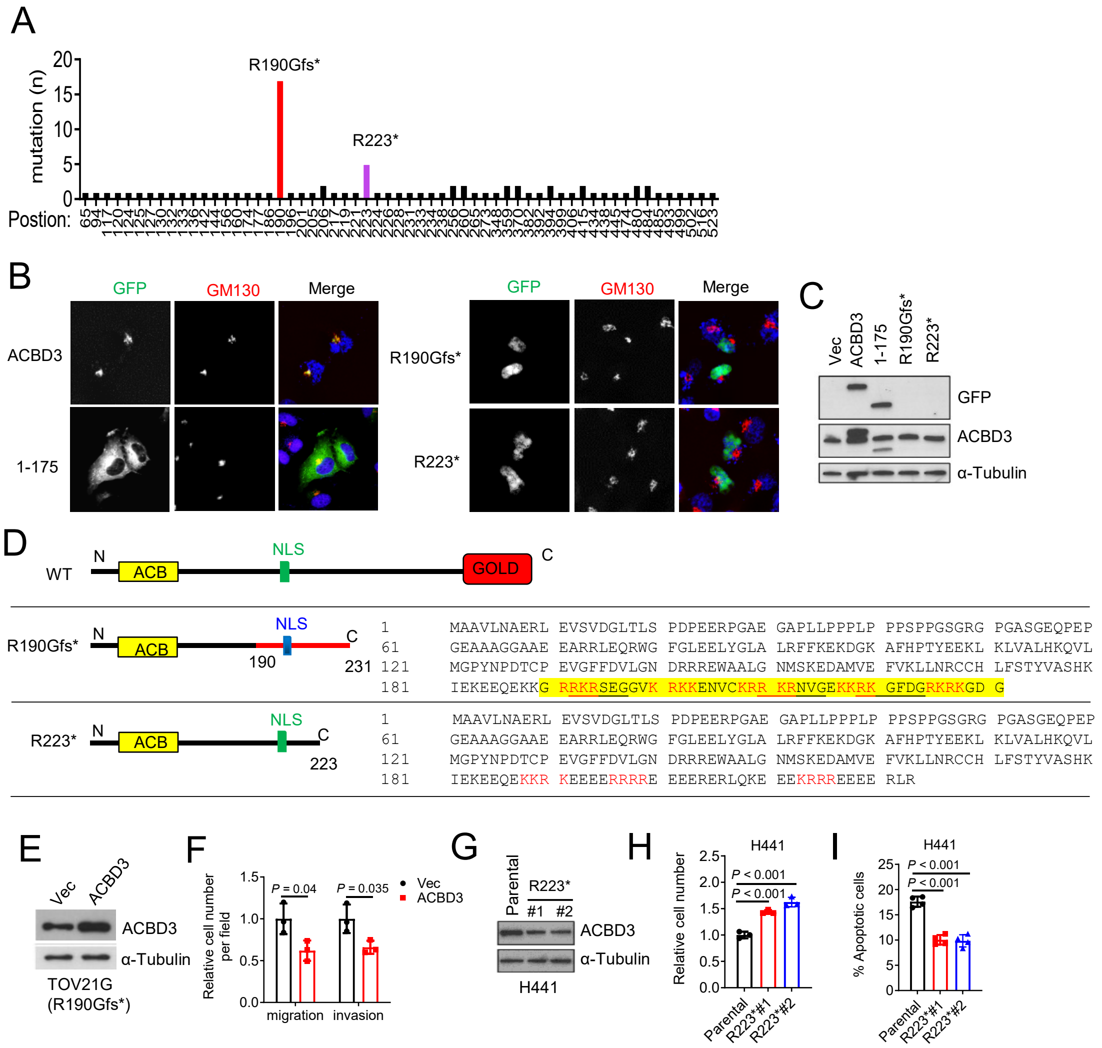

Figure S8. Loss-of-function ACBD3 mutations increase cell motility. (A) Number of ACBD3 mutations across amino acid (aa) positions (X-axis). A repeatedly occurred frameshift mutation (R190Gfs*) and nonsense mutation (R223*) were indicated. (B) Confocal images of H1299 cells transfected with the indicated ACBD3 constructs, stained with GM130 antibody and DAPI. (C) WB analysis of ACBD3 and EGFP-fused ACBD3 protein levels in H1299 cells. (D) Illustrations of ACBD3 protein aa sequences generated by R190Gfs* or R223*. Predicted nuclear localization sequences (NLS) marked in red, and newly generated aa sequences by R190Gfs* mutation highlighted in yellow. (E) WB analysis of ACBD3 protein levels in TOV21G transfectants. (F) Boyden chamber transwell migration/invasion assay of the cells from (E). (G) WB analysis of ACBD3 protein levels in parental and R223* mutant H441 cells. (H and I) Transwell migration (H) and apoptosis (I) assays of the cells from (G). Data indicate the mean ± SD from a single experiment incorporating biological replicate samples (n = 3, unless otherwise indicated) and are representative of at least 2 independent experiments. P values were determined using two-tailed Student's t-test (for F) or one-way ANOVA test (H and I).
