# Supplementary material for: Dichotomous roles of ACBD3 in NSCLC growth and metastasis
Source: Oncogene. 2025 Apr 6;44(25):2078–90. doi: 10.1038/s41388-025-03360-w (PMC12167707; doi:10.1038/s41388-025-03360-w)
Supplement: Supplementary file 2 — Supplementary table 3 [file 41388_2025_3360_MOESM2_ESM.pdf]

Table S3. Primers.

qPCR primers		
**Gene**	**Forward (5′-3′)**	**Reverse (5′-3′)**
**mRNA**		
ACBD3	TCAGTGAGTCCAGCGATGACGA	GGCACAATCTCATCCAGCAAAGG
HES1	GGAAATGACAGTGAAGCACCTCC	GAAGCGGGTCACCTCGTTCATG
HES2	AACCAGAGCCTGAGCCAGCTTA	TGCAGGAAGCGCACGGTCATTT
SNAI1	TGCCCTCAAGATGCACATCCGA	GGGACAGGAGAAGGGCTTCTC
SNAI2	ATCTGCGGCAAGGCGTTTTCCA	GAGCCCTCAGATTTGACCTGTC
HEY1	TGTCTGAGCTGAGAAGGCTGGT	TTCAGGTGATCCACGGTCATCTG
HEY2	TGAGAAGACTTGTGCCAACTGCT	CCCTGTTGCCTGAAGCATCTTC
RPL32	CCTTGTGAAGCCCAAGATCG	TGCCGGATGAACTTCTTGGT
**Constructs (pEGFP-C3)**		
ACBD3	GGAATTCTGGCGGCGCAGCT	CGGGATCCCCTGGAACAGCAGCTCTATCTAG
ACBD3-H284A	GCCAGCTGCAGGAGCAGGCCTATCAGCAGTATATGCAGCAGTT ATATC	GATATAACTGCTGCATATACTGCTGATAGGCCTGCTCCTGCAG CTGGC
PI4KB	CCGCTCGAGATGGACTACAAAGACGATGACGACAAGGGATCAG GTTCCGGGTCTATGGGAGACATGGTAGTGGAG	CGGGATCCTCACATGATGCCATTGGTG
PI4KB-VLAA	AAGGCCTGCCAGGAGGCAGCGGAGAAAGTCAAGCTTTTGCAT	ATGCAAAAGCTTGACTTTCTCCGCTGCCTCCTGGCAGGCCTT
**Constructs (pLVX)**		
ACBD3	GGAATTCATGGCGGCGCAGCT	CGGGATCCCCTGGAACAGCAGCTCTATCTAG
**Constructs (pEGFP-C3-MAO)**		
ACBD3-MAO	GGAATTCTGGCGGCGCAGCT	CGGGATCCCTAGTATAATAGACTCTGTAGTAGACGGACTTG
ACBD3 (1-505)-MAO	GGAATTCTGGCGGCGCAGCT	CGGG ATCCTTGAGGAGATAGACTCCCCTCCC
ACBD3 (1-393)-MAO	GGAATTCTGGCGGCGCAGCT	GGGGTACCATCCTGCTGAATCTTCTCTTTGA
ACBD3 (1-173)-MAO	GGAATTCTGGCGGCGCAGCT	CGGG ATCCTTGAGGAGAGGACAACACTTATTTAGAAG
ACBD3 (1-78)-MAO	GGAATTCTGGCGGCGCAGCT	CGGGATCCTTGCCAAAGCCCCAGTGC
ACBD3 (174-393)-MAO	GGAATTCTGTCGGCATATGTTGCGTCC	GGGGTACCATCCTGCTGAATCTTCTCTTTGA
ACBD3 (174-249)-MAO	GGAATTCTGTCGGCATATGTTGCGTCC	CGGG ATCCTTGTCTTTGATTTGTGGTCTTGTCC
ACBD3 (250-393)-MAO	GGAATTCTGACTGCCGTGCAATTCCA	GGGGTACCATCCTGCTGAATCTTCTCTTTGA
**Constructs (TurboID)**		
PI4K2A	CCGCTCGAGATGTACCCATACGATGTTCCAGATTACGCTATGGA CGAGACGAGCCC	GCTCTAGACTACCACCATGAAAAGAAGGG
PI4KB	CCGCTCGAGATGGACTACAAAGACGATGACGACAAGGGATCAG GTTCCGGGTCTATGGGAGACATGGTAGTGGAG	GCTCTAGATCACATGATGCCATTGGTG
